# Higher modified dietary inflammatory index is associated with increased risk of osteoporosis in US adults: Data from NHANES

**DOI:** 10.3389/fnut.2022.891995

**Published:** 2022-08-09

**Authors:** Yong Chen, Fu-hua Chen, Yi-qing Chen, Qiu Zhang

**Affiliations:** Department of Endocrinology, First Affiliated Hospital of Anhui Medical University, Hefei, China

**Keywords:** osteoporosis, dietary inflammatory index, NHANES, American, nutrition

## Abstract

**Objective:**

The aim of this study was to study the relationship between modified dietary inflammatory index (MDII) score with osteoporosis (OP) in adult Americans.

**Methods:**

Data were extracted from the United States National Health and Nutrition Examination Survey (NHANES) (2007–2008, 2009–2010, 2013–2014, and 2017–2018). In this cross-sectional study, 5,446 participants were included and analyzed. Potential dietary inflammatory was assessed by MDII score (24-h recall), a composite method computed according to the relationship between nutrients and systemic pro-inflammatory cytokine level, and was further classified into tertiles. Weighted multivariable logistic regression analysis was employed to examine the associations between OP and MDII scores.

**Results:**

In weighted multivariable-adjusted logistic regression models, the highest tertile of MDII score was associated with an increased risk of OP [odds ratio (OR): 1.73, 95% confidence interval (95 CI%): 1.14–2.63]. In participants aged above 59 years, a higher MDII score showed a higher risk of OP (OR: 1.92; 95 CI%: 1.16–3.15). In the sex-stratified models, the results remained significant only among women (OR: 1.80; 95% CI: 1.02–3.17). In the menopausal status stratified model, after adjusting potential confounding variables, the association between the MDII score, either as a categorical (OR: 1.88; 95% CI: 1.07–3.13) or continuous variables (OR: 1.19; 95%CI: 1.02–1.38), and OP risk was significant among postmenopausal women.

**Conclusion:**

Our study indicates that a higher MDII score (pro-inflammatory effect) is significantly associated with an increased risk of OP in US adults, especially among those postmenopausal women more than 60 years. This study further supports that those dietary changes have the potential to prevent OP.

## Introduction

In the United States, the prevalence of osteoporosis (OP) in 2017–2018 at the femur neck, lumbar spine, or both sites among adults above 50 years was 12.6%; 19.6% among women; and 4.4% among men (1). OP is caused by many factors and results in low bone mass and degradation of bone microstructure, which increases the risk of fractures (2). OP-related fractures decrease the quality of life of patients and impose a heavy economic burden on the healthcare system. Regarding the total direct costs associated with OP care, the fracture treatment and drug costs were €37.4 billion in 2010, which increased by 64% to €56.9 billion in 2019 (3, 4). This result was consistent with a 50% increase in the costs of the treatment of fractures by 2025, as predicted by Burge et al. who studied and estimated the fracture-treatment costs in the USA from 2005 to 2025 (5). Circulating levels of inflammatory markers can predict bone loss and bone resorption in older adults to some extent (6). The presence of serum inflammatory markers worsens the destruction of bone and increases the risk of fractures in older people (7–9).

The risk factors for OP are multifactorial, including sex, diet, race, lifestyle, genetics, advanced age, low calcium intake, smoking, and alcohol consumption, which might promote the progression of OP (10). There are four main dietary patterns in relation to bone health (i.e., Mediterranean, Eastern, Western, and Modern diets). The Mediterranean diet showed a positive relationship with higher bone mass among premenopausal women, lower incidence of hip fracture in the elderly, and low risk of falling among older people (11–13). Compared with studies on the effects of other dietary patterns, studies on the effects of the Eastern diet (ED) are limited. As the main feature of ED, the effect of high carbohydrates on the regulation of bone health is debatable (14, 15). Coxam reported that postmenopausal women who were administered a diet enriched in phytoestrogens (nutrients found in the ED and beneficial to the bones) had a lower risk of fracture due to OP (16). Western diets (WDs) are considered to be unhealthy. Four aspects are related to the adverse effects of the WD on bone health. (1) Due to the deficiency of dietary potassium and alkaline minerals in the WD, alkaline salts (such as calcium and magnesium phosphates) are released from the bone to balance the systemic pH, which destroys the bone and increases the risk of fracture (17). (2) The WD contains large quantities of saturated FAs, which adversely affect bone health. (3) Due to the low intake of fruits, vegetables, and whole grains, the level of dietary fiber, which positively affects bone health, is low in the WD. (4) Highly processed foods constitute the WD, provide large quantities of fat and sugar, and can adversely affect bone health. Modern diets (MDs) comprise highly processed foods. Processed foods are not only convenient and nutritious but also improve bioavailability of isoflavones, the utilization of minerals, and enhance the production of MK-7. However, they contain large quantities of phosphorus, sodium, and “hidden” sugars, which are associated with OP (18, 19).

The Dietary Inflammation Index (DII) was developed to evaluate the inflammatory potential of a diet; a higher DII score indicates a pro-inflammatory diet (2). A study in Korea has reported a significant association between a higher DII score and higher OP risk in women (20). However, a study from the Brazilian Osteoporosis Study (BRAZOS) database reported a lack of association between the DII score and low-impact fractures in the Brazilian population (21). A cohort study from China reported a positive association between pro-inflammatory diets and an increase in the long-term risk of OP in older Chinese women (22). Consistent with a previous study, the above studies proved that bone mineral density differs with race, ethnic group, and diet (23). Therefore, determining the association between OP and modified dietary inflammatory index (MDII) might provide information for the prevention and treatment of OP among different races. To the best of our knowledge, the relationship between MDII score and OP in the United States has not been studied. In this study, the data of 5,446 participants were extracted from the National Health and Nutrition Examination Survey (NHANES) to determine the association between dietary inflammation and OP.

## Methods

### Data source and study design

**Figure 1:**
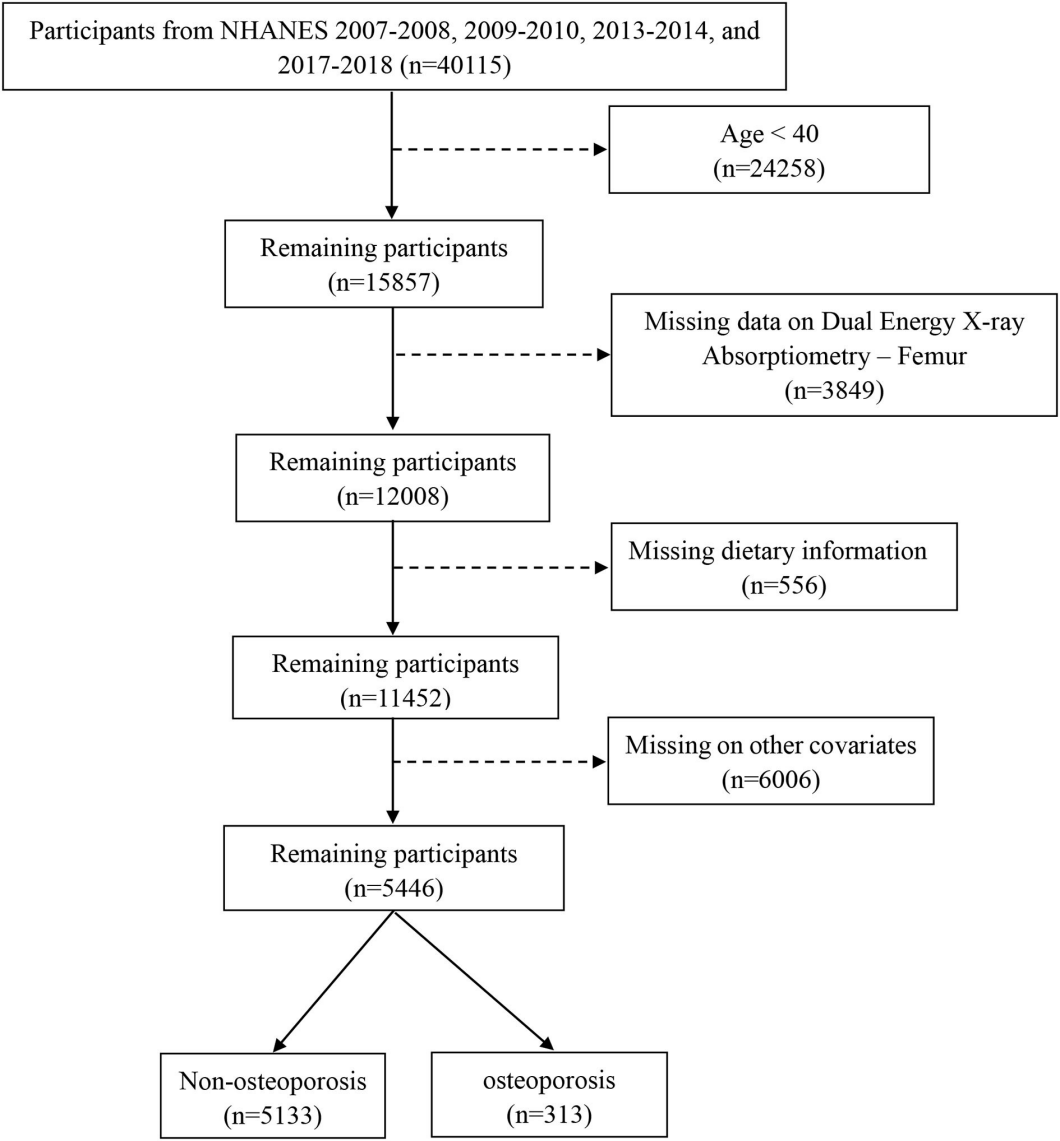
The flowchart of this study. Data from NHANES 2007–2018 survey cycle, except for 2011–2012 and 2015–2016. Analysis was restricted to adults >40 years of age with an examination for dual-energy X-ray absorptiometry femur bone data and a dietary interview. Exclusion criteria included those under 40 years, missing data on dual-energy X-ray Absorptiometry-Femur, dietary information, and incomplete other covariates.

The data for this analysis were obtained from NHANES, which is a cross-sectional investigation designed to assess the health and nutritional status of both adults and children in the United States. Details of the survey design have been reported previously (24). This study was restricted to participants aged above 40 years who had an examination for dual-energy X-ray absorptiometry femur bone data and a dietary interview from 2007 to 2018 in the NHANES database, except for the 2011–2012 and 2015–2016 survey cycles in which there are no data related to OP, with complete data on other covariates needed in this study. The detailed inclusion and exclusion in this study are shown in Figure 1.

### Definition of OP

The BMD of the participants was measured using a Hologic QDR-4500A fan-beam densitometer (Hologic, Inc., Bedford, Massachusetts). Detailed information on the DXA examination is accessible on the NHANES website (25). In this study, we mainly analyzed the femur neck BMD due to its highest predictive value for hip fracture, and the hip is the site of high clinical relevance (26). The diagnosis of OP was based on T-score results and self-reported. T-score was calculated as (BMD_mesured_ – mean BMD_reference_)/SD_reference_. OP was defined as a T-score of BMD ≦ −2.5 (27). Consistent with the International Society for Clinical Densitometry's corresponding guidelines, the reference group for calculating T-score for the femur neck is composed of 20–29 years old non-Hispanic Caucasian participants from NHANES III (28).

### Modified dietary inflammatory index

In this study, data on dietary intake were obtained from one in-person 24-h diet recall interview (24-h recall). The 24 h-derived dietary data were used to calculate the MDII score for all included participants. A complete description of the DII is available elsewhere (29). In brief, the dietary intake data are estimated from the types and amounts of foods and beverages (including all types of water) consumed during the 24-h prior to the interview. In this study, 28 of the 45 food parameters were calculated, namely, energy, carbohydrate, protein, fiber, total fat, cholesterol, saturated fatty acids, monounsaturated fatty acids, polyunsaturated fatty acids, n-3 fatty acids, n-6 fatty acids, alcohol, caffeine, beta carotene, vitamin A, vitamin B6, vitamin B12, vitamin B1, vitamin B2, vitamin C, vitamin D, vitamin E, iron, magnesium, zinc, selenium, niacin, and folic acid (Table 1). To calculate the modified DII score, the dietary intake of each food parameter is subtracted from the global daily mean intake, dividing by its standard deviation, converting the value to a percentile score, doubling each percentile score, and subtracting “1” to achieve a symmetrical distribution. Then, the intake data were multiplied by the respective inflammatory score for each food parameter. By summing each food parameter-specific DII score, we can obtain an individual overall modified DII score. To study the relationship between the modified DII score and OP, we further divided the modified DII score into tertiles with the following cut points: T1: (−4.870 to 0.419); T2: (0.4191–2.015); and T3: (2.016–4.577).

**Table 1:** Components of the dietary inflammatory index (DII) in this study.

	**DII component**	**Overall inflammatory effect score^a^**
1.	Energy (kcal)	0.18
2.	Protein (g)	0.021
3.	Carbohydrate (g)	0.097
4.	Dietary fiber (g)	−0.663
5.	Total fat (g)	0.298
6.	Total saturated fatty acids (g)	0.373
7.	Total monounsaturated fatty acids (g)	−0.009
8.	Total polyunsaturated fatty acids (g)	−0.337
9.	Cholesterol (mg)	0.110
10.	Vitamin E (mg)	−0.419
11.	Vitamin A (μg)	−0.401
12.	Beta-carotene (μg)	−0.584
13.	Thiamin (Vitamin B1) (mg)	−0.098
14.	Riboflavin (Vitamin B2) (mg)	−0.068
15.	Niacin (mg)	−0.246
16.	Vitamin B6 (mg)	−0.365
17.	Folic acid (μg)	−0.190
18	Vitamin B12 (μg)	0.106
19.	Vitamin C (mg)	−0.424
20.	Vitamin D (D2 + D3) (μg)	−0.446
21.	Magnesium (mg)	−0.480
22.	Iron (mg)	0.032
23.	Zinc (mg)	−0.313
24.	Selenium (μg)	−0.91
25.	Caffeine (mg)	−0.110
26.	Alcohol (g)	−0.278
27.	n-3 fatty acids (g)	−0.436
28.	n−6 fatty acids (g)	−0.159

^a^*Overall inflammatory effect score refers to the “food parameter-specific overall inflammatory effect score” accounting for the robustness of the literature, which is considered optimal at the median of 236 articles. Taking dietary fiber as an example, its score represents an anti-inflammatory effect, while total fat represents a pro-inflammatory effect*.

### Covariates

Demographic factors (i.e., age, race, sex, education, and poverty level index), physical activity, drinking history, smoking status, hypertension status, diabetes classification, history of fracture, history of prednisone or cortisone, menopausal status, and calcium intake were obtained from NHANES. The age was categorized as 40–59 and ≥60 years. The races were categorized as Mexican American, non-Hispanic White, non-Hispanic black other Hispanic, and other Races. Education was categorized as less than high school graduate; high school graduate/GED; and above high school graduate. The poverty level index was classified as monthly poverty level index ≦ 1.30; 1.30 < monthly poverty level index ≦ 1.85; and monthly poverty level index > 1.85. Physical activity was classified as inactive group (no leisure-time physical activity), insufficiently active group (leisure-time moderate activity 1–5 times per week with metabolic equivalents ranging from 3 to 6 or leisure-time vigorous activity 1–3 times per week with metabolic equivalents >6), or active group (those who had more leisure-time moderate or vigorous activity than above) (30). Drinking history was defined as one who has ever had 1 drink of any alcoholic beverages, including liquor, beer, wine, wine coolers, and any other type of alcoholic beverage in his/her entire life, not counting small tastes or sips. Smoking status was defined as current smokers who smoked >100 cigarettes in their lifetime and smoked cigarettes at the time of investigation; former smokers who had smoked >100 cigarettes during their lifetime and quit smoking before the time of the survey. The diabetes classification in this study was based on the Standards of Medical Care in Diabetes-2021 (31). Participants were divided into three groups, namely, normal (HbA1c ≤ 5.6% and without self-reported diabetes), prediabetes (HbA1c 5.7–6.4%), and diabetes (HbA1c ≥ 6.5%, fasting glucose ≥ 7 or self-reported diabetes). Hypertension status (yes or no), history of hip, wrist, or spine fracture (yes or no) were also extracted from dataset. Menopausal status was classified as pre/peri-menopausal (women who had regular periods in the past 12 months) or postmenopausal (who had no regular periods in the past 12 months).

### Statistical analysis

The overall statistical analysis in this study was performed according to the analytic guidelines suggested by the centers for Disease Control and Prevention (CDC) (32). Categorical variables were presented as frequencies (percentages), while continuous variables as mean (standard error, SE). Notably, MDII score was analyzed as a continuous and categorical variable. Rao-Scott χ^2^ test was adopted for categorical variables and svyttest for continuous variables with RStudio.

Odds ratios (ORs) and 95% confidence intervals (CIs) for risk of OP among all participants and participants stratified by age, sex, and postmenopause were estimated by weighted multivariable logistic regression. Model 1 was adjusted for age and sex, and model 2 further for model 1 plus hypertension, smoking status, physical activity, history of fracture, DM, race, BMI, education, history of prednisone or cortisone, calcium intake, alcohol use, and poverty index.

All analyses in this study were conducted using RStudio version 1.4.1717. All tests were two-tailed, and statistical significance was set at *P* < 0.05.

## Results

### Baseline characteristics of participants

Following exclusion of individuals with incomplete bone health data, other covariates, or dietary recall assessments, data from 5,446 participants of 4 circles NHANES, 2007–2008, 2009–2010, 2013–2014, and 2017–2018, aged ≥ 40 years were analyzed (Figure 1). According to the criteria used to define OP in this study, 313 participants were identified as OP. The demographics and baseline characteristics of this study are presented in Table 1. OP was more prevalent in older women (≥60). Women had a higher proportion of OP than men (68.4 vs. 31.6). There were significant differences between the two groups in age, race, BMI, physical activity, drinking history, hypertension, history of fracture, history of prednisone or cortisone, and menopausal status of women. In addition, all 5,446 participants were categorized into three groups according to MDII tertiles: T1 (−4.870 to 0.419), T2 (0.4191–2.015), and T3 (2.016–4.577). Individuals with OP showed to have a higher proportion in the highest DII score (T3) than any other OP group (Table 2). Table 3 demonstrates that women had a higher DII score than men (*P* < 0.001), and Non-Hispanic Black is more likely to have a higher DII score. However, there was no difference in DII scores between age groups.

**Table 2:** Characteristics of the study population.

	**Non-osteoporosis (*N* = 5,133)**	**Osteoporosis (*N* = 313)**	***P*-value**
Age, *n* (%)			<0.001
40–59	2,966 (57.8)	56 (18.2)	
≥60	2,167 (42.2)	257 (81.8)	
Sex, *n* (%)			<0.001
Male	2,613 (50.9)	99 (31.6)	
Female	2,520 (49.1)	214 (68.4)	
Race/ethnicity, *n* (%)			<0.001
Mexican American	380 (7.4)	12 (3.9)	
Non-Hispanic White	3,603 (70.2)	255 (81.5)	
Non-Hispanic Black	601 (11.7)	11 (3.5)	
Other Hispanic	241 (4.7)	10 (3.2)	
Other Race	308 (6)	25 (7.8)	
BMI, *n* (%)			<0.001
<18.5	51 (1)	11 (3.4)	
18.5–24.9	1,067 (20.8)	141 (44.9)	
25.0–29.9	1,858 (36.2)	97 (30.9)	
≥30.0	2,157 (42)	64 (20.8)	
Education, *n* (%)			0.332
Less than high school graduate	1,026 (20)	73 (23.2)	
High school graduate/GED	1,263 (24.6)	86 (27.6)	
Above high school graduate	2,844 (55.4)	154 (49.1)	
Poverty level index, *n* (%)			0.067
Monthly poverty level index ≤ 1.30	1,099 (21.4)	85 (27)	
1.30 < Monthly poverty level index ≤ 1.85	708 (13.8)	55 (17.7)	
Monthly poverty level index > 1.85	3,326 (64.8)	173 (55.4)	
Physical activity, *n* (%)			<0.001
Inactive	3,783 (73.7)	275 (87.9)	
Insufficiently active	703 (13.7)	18 (5.8)	
Active	647 (12.6)	20 (6.3)	
Drinking history, *n* (%)			0.001
Yes	4,060 (79.1)	206 (65.8)	
No	1,073 (20.9)	107 (34.2)	
Smoking status, *n* (%)			0.349
Non-smoker	2,613 (50.9)	161 (51.5)	
Former smoker	955 (18.6)	70 (22.4)	
Current smoker	1,565 (30.5)	82 (26.1)	
Diabetes classification, *n* (%)			0.214
Diabetes	1,976 (38.5)	85(27.2)	
Pre-diabetes	1,283 (25)	132 (42.2)	
Normal	1,874 (36.5)	96 (30.1)	
Hypertension, *n* (%)			<0.001
Yes	2,654 (51.7)	213(68.1)	
No	2,479 (48.3)	100 (31.9)	
History of fracture, *n* (%)			<0.001
Yes	873 (13.7)	82 (26.2)	
No	4,430 (86.3)	231 (73.8)	
History of prednisone or cortisone, *n* (%)			
Yes	359 (7)	25 (8.1)	0.552
No	4,774 (93)	288 (91.9)	
Menopausal status, *n* (%)			<0.001
Pre/peri-	502 (20.9)	3 (1.4)	
Post-	1,895 (79.1)	211 (99.6)	
Calcium intake, SE	929 (12.6)	866.1 (66.2)	0.345
Modified dietary inflammatory index, *n* (%)			0.018
T1 (−4.870 to 0.419)	1,879 (36.6)	85 (27)	
T2 (0.4191–2.015)	1,714 (33.4)	99 (31.5)	
T3 (2.016–4.577)	1,540 (30.1)	129 (41.5)	

*SE, standard error*.

**Table 3:** Characteristics of osteoporosis (OP) participants by modified dietary inflammatory index (MDII) score.

	**Dietary inflammatory Index score (continuous)**		***P*-value**
	**Mean**	**SE**	
Sex			<0.001
Male	0.67	0.05	
Female	1.15	0.05	
Age			0.09
40–59	0.85	0.06	
≥60	0.98	0.05	
Race/ethnicity			<0.001
Mexican American	0.82	0.08	
Non-Hispanic White	0.86	0.06	
Non-Hispanic Black	1.25	0.07	
Other Hispanic	0.97	0.10	
Other Race	0.91	0.15	

*SE, standard error*.

### Association between MDII and OP

**Figure 2:**
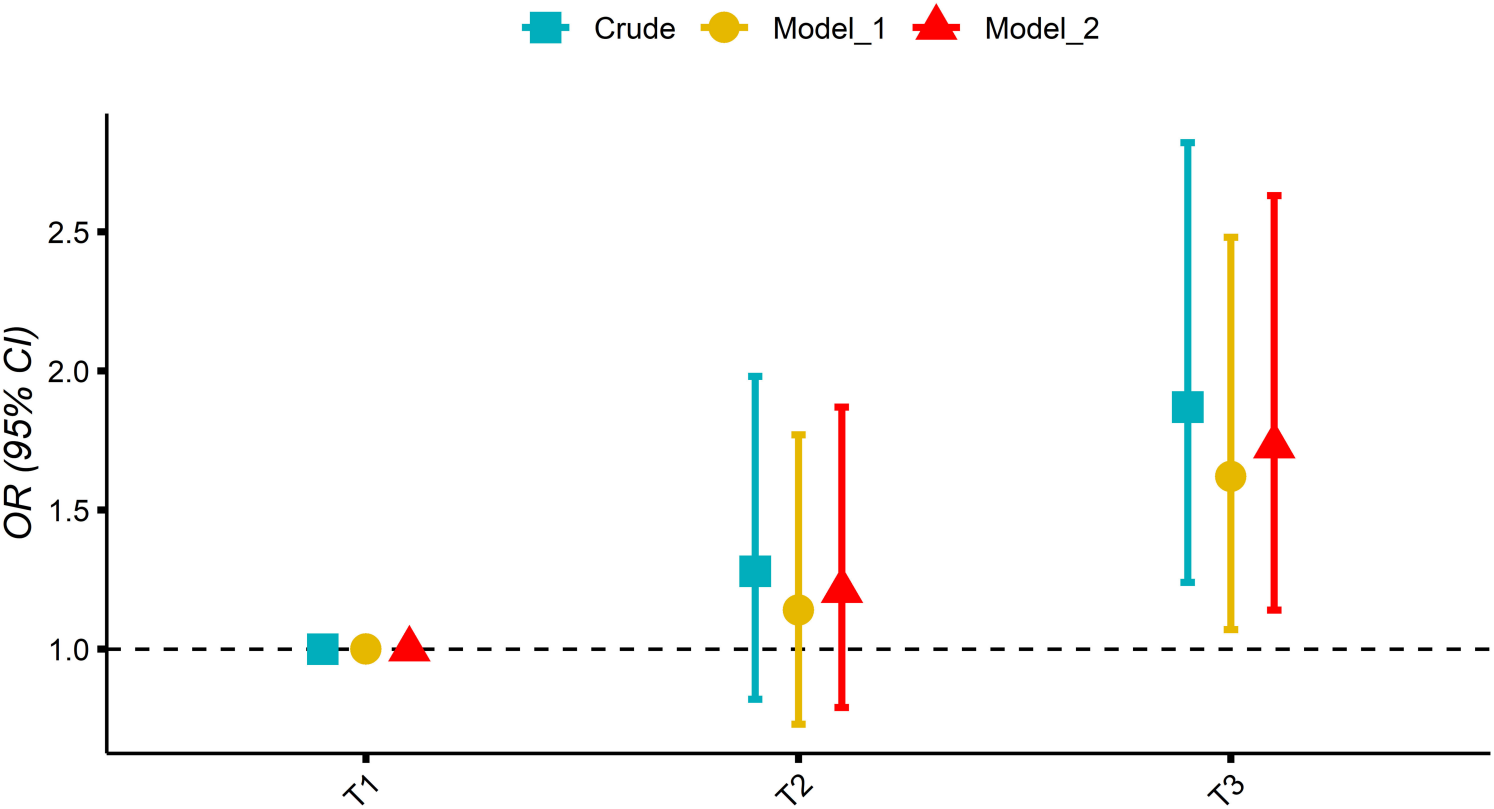
Association of tertile of the modified dietary inflammatory index (MDII) score with osteoporosis (OP) in participants; mode 1 adjusted: age and sex; model 2 adjusted: model 1 plus hypertension, smoking status, physical activity, history of fracture, DM, race, BMI, education, history of prednisone or cortisone, calcium intake, alcohol use, and poverty index.

Figure 2 shows the association of MDII score with OP in participants. While taking the lowest tertiles of MDII score (T1) as a reference, the weighted logistic regression models showed a significantly increased OR of OP for the participants in the highest tertile of MDII score after adjusting for potential confounding factors (T2: OR 1.21, 95% CI: 0.79–1.87; T3: OR 1.73, 95% CI: 1.14–2.63; and *P* for trend = 0.013).

### Association between MDII and OP stratified by age and sex

As is shown in Table 4, the highest tertile of MDII in the individuals 60 years group had a significantly higher OR of OP than the lowest one after adjusting potential confounding factors (OR: 1.92; 95% CI: 1.16–3.15; *P* = 0.01). However, no significant association between MDII score and OP risk was observed in the subgroup of those participants aged between 40 and 59 years (OR: 1.18; 95% CI: 0.45–3.1; *P* = 0.73). Women who had a higher MDII score had a significantly higher OR for risk of OP (OR: 1.80; 95% CI: 1.02–3.17; *P* = 0.043), while there was no significant association between MDII and OP risk in men (OR: 1.63; 95% CI: 0.74–3.62; *P* = 0.22).

**Table 4:** Multivariate logistic model for OP risk stratified by age and sex.

	**Age**				**Sex**			
	**40** ≦**Age**<**60**		**Age** ≥**60**		**Male**		**Female**	
**MDII**	**OR (95%CI)**	***P*** **-value**	**OR (95%CI)**	***P*** **-value**	**OR (95%CI)**	***P*** **-value**	**OR (95%CI)**	***P*** **-value**
T1	Reference^a^		Reference^a^		Reference^b^		Reference^c^	
T2	0.617 (0.20–1.73)	0.35	1.35 (0.85–2.13	0.19	0.81 (0.47–1.41)	0.45	1.58(0.84–2.96)	0.15
T3	1.18 (0.45–3.1)	0.73	1.92 (1.16–3.15)	0.01	1.63 (0.74–3.62)	0.22	1.80 (1.02–3.17)	0.043
*P* for trend		0.605		0.013		0.307		0.045

^a^*Multivariable adjusted: sex, hypertension, smoking status, physical activity, history of fracture, DM, race, BMI, education, history of prednisone or cortisone, calcium intake, alcohol use, and poverty index*.

^b^*Multivariable adjusted: age, hypertension, smoking status, physical activity, history of fracture, DM, race, BMI, education, history of prednisone or cortisone, calcium intake, alcohol use, and poverty index*.

^c^*Multivariable adjusted: age, menopausal status, hypertension, smoking status, physical activity, history of fracture, DM, race, BMI, education, history of prednisone or cortisone, calcium intake, alcohol use, and poverty index*.

### Association between MDII and OP stratified by postmenopausal participants

**Figure 3:**
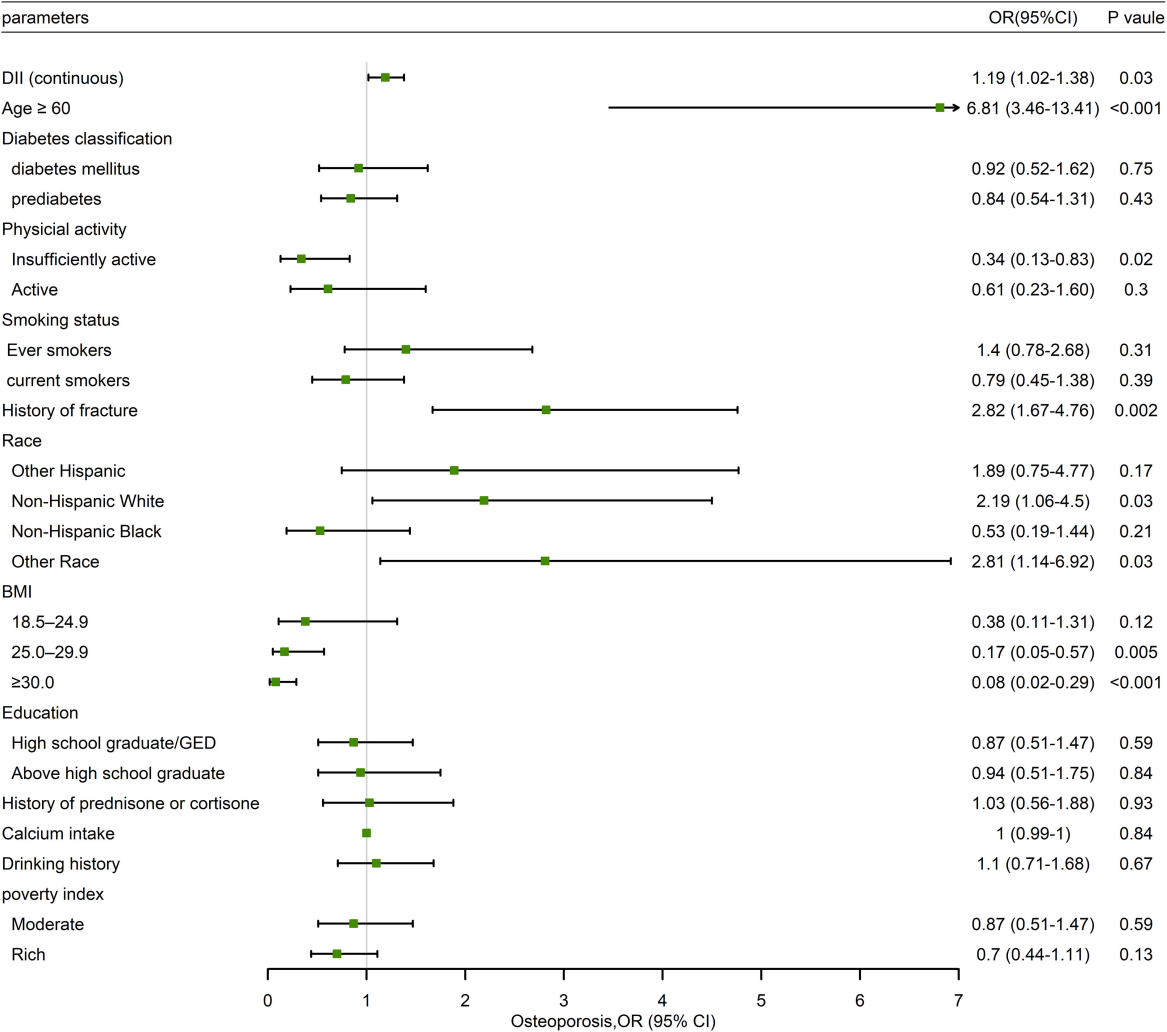
Forest plot of weighted multivariable logistic regression analysis model in participants demonstrating the association between MDII score and OP.

In Table 5, the associations between the MDII and OP, stratified by postmenopausal status, were also determined. In the unadjusted model, the highest tertile of MDII had a higher risk of OP than the lowest one (OR: 2.01; 95% CI: 1.24–3.23; *P* = 0.005). Similarly, after adjusting potential confounding variables, the association between the MDII, either as a categorical (OR: 1.88; 95% CI: 1.07–3.13; *P* = 0.03) or continuous variables (OR: 1.19; 95% CI: 1.02–1.38; *P* = 0.03), and OP risk remained significant among postmenopausal female participants (Figure 3). As is shown in Figure 3, in the forest plot, insufficient activity, and higher BMI are protective factors for OP, while old age, non-Hispanic White, other races, and MDII score are risk factors among postmenopausal women.

**Table 5:** Association of MDII score and OP risk in postmenopausal female participants.

	**Crude**		**Multivariable adjusted** ^**a**^	
	**OR (95%CI)**	***P*-value**	**OR (95%CI)**	***P*-value**
MDII (continuous)	1.18 (1.05–1.32)	0.006	1.19 (1.02–1.38)	0.03
**MDII (tertiles)**
T1	Reference		Reference^a^	
T2	1.55 (0.86–2.72)	0.14	1.63 (0.86–3.07)	0.13
T3	2.01 (1.24–3.23)	0.005	1.88 (1.07–3.13)	0.03
*P* for trend		0.005		0.008

^a^*Multivariable adjusted: age, hypertension, smoking status, physical activity, history of fracture, DM, race, BMI, education, history of prednisone or cortisone, total calcium intake, alcohol use, and poverty index*.

## Discussion

This is the first study on the association between MDII score and OP in US adults. In our study, higher MDII was significantly related to OP. Among women, the MDII score had a significant association with the risk of OP, and participants with the highest MDII score had a high risk (80%) of OP after adjusting for potential confounding factors. However, the association between MDII score and OP among men was not statistically significant. These results were similar to those reported by Kim et al. in the KoGES_Health Examinee (HEXA) cohort study (20). This might be due to the difference in hormone status and genetic differences between men and women, and autoimmune diseases such as OP are common in women (33, 34).

A strong relationship between OP and inflammation was found in many studies (35–37). Chronic inflammation is an important risk factor for OP and adversely affects bone formation (38). Specific dietary patterns such as MD, WD, ED, and modern diets significantly affect the levels of inflammation (18, 39, 40). For example, representative dietary nutrients, such as fatty acids, proteins, bioactive peptides, phytoestrogens, and prebiotics can promote or inhibit inflammation-related bone health (41–45). Potential adverse effects of a pro-inflammatory diet on bone mineral density were reported in a study (46). Therefore, we hypothesized that the MDII score would be a reliable indicator for predicting the inflammatory response of OP among adults in the US. In this study, the MDII score of the elderly was positively associated with the diagnosis of OP. This might be because nutrients in the circulatory system of individuals of different ages can directly affect bone mineral density (47). Additionally, unlike younger individuals, the elderly have different gut microbial communities, which may affect the metabolism of the host under the influence of diverse dietary patterns (48, 49).

Some studies have reported the association between MDII score and bone health among postmenopausal women. A small cross-sectional study in Iran demonstrated women with higher DII scores had lower BMD in the lumbar spine but not in the femoral neck (50). However, a report on the NAHNES samples from adults in the US showed that a higher DII score in both men and women was related to a lower mean level of BMD in the total femur and femoral neck (46). Similar to our results, a recent study reported that higher DII scores were related to a lower BMD and higher risk of OP in postmenopausal Korean women (51). Therefore, the association between MDII score and OP in postmenopausal women differs with race and age; further studies regarding this need to be performed.

The underlying mechanism of the association between the DII score and bone health has been discussed in previous studies. Among the food parameters included in the MDII, fiber, β-carotene, riboflavin, flavones, and vitamins A, C, D, and E,n-3 and n-6 fatty acids mainly exhibit anti-inflammatory effects, while total fat, trans fat, and cholesterol are the main pro-inflammatory properties (29). OP is a chronic inflammatory response, considering that some pro-inflammatory markers, such as tumor necrosis factor (TNF-a), interleukin (IL)-1b, and IL-6 can regulate RANKL expression, which increases the activity of osteoclast (52, 53). Due to uncertainties regarding the association between calcium and inflammation, calcium intake was not considered while calculating the MDII score (29). Although calcium intake was not significantly different between non-OP and OP groups in our study, calcium is the main factor that helps to maintain bone health and is a major component of the bone matrix. Thus, we included calcium intake in our multivariable model as an independent variable determining the association of MDII score and OP, and the results remained significant.

This study has several limitations. First, this was a cross-sectional study, and therefore, causation could not be deduced from the results. Second, although self-reported 24-h dietary recall data are the most practical and useful method in the NHANES, recall bias might occur due to overreporting or underreporting. In addition, only 28 of the 45 food parameters were incorporated while calculating the MDII score. However, previous studies have proved that even if the numbers of food parameters used for the MDII score are below 30, the MDII score is still convincing (54, 55). Finally, many participants were excluded due to missing data on selected covariates, which prevented our results from reflecting a nationwide pattern. Despite these limitations above, our study had several advantages. First, it is the first study to investigate the association between MDII score and OP risk among adults in the US. Second, the potential confounding factors were adjusted, and the MDII score was treated both as a categorical and continuous variable, which increased the reliability of the findings concerning the subgroup of postmenopausal women. Third, using a relatively large and nationally representative database to determine the relationship between inflammation of diet and OP is the crucial strength of this study.

## Conclusion

The results of this study suggested a significant association of a pro-inflammatory diet and an increase in the risk of OP in the elderly (≥60 years) and women. Additionally, postmenopausal women with higher MDII scores had a higher risk of OP. These findings may suggest that individuals who have a high risk of OP (e.g., women, postmenopausal women, and the elderly) need dietary advice.

## Data availability statement

The raw data supporting the conclusions of this article will be made available by the authors, without undue reservation.

## Ethics statement

The studies involving human participants were reviewed and approved by National Center for Health Statistics Research Ethics Review Board. The patients/participants provided their written informed consent to participate in this study.

## Author contributions

QZ and YC contributed to the conception of this study. F-hC and Y-qC contributed to analysis and manuscript preparation. YC performed the data analyses and drafted the manuscript. All authors contributed to the article and approved the submitted version.

## Conflict of interest

The authors declare that the research was conducted in the absence of any commercial or financial relationships that could be construed as a potential conflict of interest.

## Publisher's note

All claims expressed in this article are solely those of the authors and do not necessarily represent those of their affiliated organizations, or those of the publisher, the editors and the reviewers. Any product that may be evaluated in this article, or claim that may be made by its manufacturer, is not guaranteed or endorsed by the publisher.
